# Analysis of Specific IgG Titers Against Tick-Borne Encephalitis in Patients with Primary Antibody Deficiency Under Immunoglobulin Substitution Therapy: Impact of Plasma Donor Origin

**DOI:** 10.3389/fimmu.2014.00675

**Published:** 2015-01-05

**Authors:** Sigune Goldacker, Torsten Witte, Daniela Huzly, Michael Schlesier, Hans-Hartmut Peter, Klaus Warnatz

**Affiliations:** ^1^Center for Chronic Immunodeficiency, University Medical Center and University of Freiburg, Freiburg, Germany; ^2^Clinic for Immunology and Rheumatology, Hannover Medical School, Hannover, Germany; ^3^Institute for Medical Microbiology & Hygiene, University Medical Center Freiburg, Freiburg, Germany

**Keywords:** tick-borne encephalitis, primary antibody deficiency, CVID, IVIG, SCIG, passive immunization

## Abstract

Immunoglobulin (Ig) replacement therapy is effective in reducing infections in patients with primary antibody deficiency (PAD). Diversity of specific antibodies is achieved by pooling plasma from over 1000 donors usually of a given geographic region. However, there is no agreement with regard to an optimal vaccination schedule for plasma donors. Especially for tick-borne encephalitis (TBE), regional vaccination rates differ widely among populations due to the epidemiology of the disease. We analyzed specific antibody titers against TBE in comparison to total IgG levels in 162 serum samples collected from 110 PAD patients substituted with polyvalent intravenous IgG or subcutaneous IgG. Some patients received different IgG products over time leading to a total number of 122 different patient-IgG product combinations. Positive TBE-specific IgG levels were detected in 35 cases when measured by standard ELISA and could be confirmed by demonstration of neutralizing antibodies in 31 cases. The detection of specific antibody levels correlated with the geographic origin of the IgG preparations. No titers were detectable in patients substituted with IgG products from North-American donors, whereas variable degrees of anti-TBE titers were observed in patients receiving products from different European countries. We suggest considering the patients’ personal risk for TBE when selecting an appropriate Ig preparation. These data support regional plasma donation in order to address the diverse local infection profile.

## Introduction

Distribution of tick-borne encephalitis (TBE) is known to show immense geographic differences leading to variable need for vaccination-induced protection of individuals (1). Patients suffering from primary antibody deficiency (PAD) are characterized by reduced or absent antibody responses following vaccination^1^. They depend on continuous Immunoglobulin G (IgG) replacement therapy to maintain a diverse antibody repertoire. Plasma-producing companies recruit plasma donors globally and the origin of plasma donors varies considerably in between commercially available IgG products even between different batches of the same product. The proof of representative specific antibody titers within the products is obligatory for authorization of each batch. Quality management within the companies often provides data for an even broader spectrum of specific antibodies, but usually not anti-TBE IgG titers. Rabel et al. reported 2012 geographic variation of neutralizing antibodies against TBE within intravenous IgG preparations (2). Seidel et al. mention passive transfer of protective anti-TBE IgG levels via IgG replacement therapy in their publication focusing on active TBE vaccination responses in 18 patients (3). However, protective antibody levels within patients have not been systematically studied so far. Epidemiological data on the prevalence of TBE infection within PAD patients do not exist, so it can only be speculated that PAD patients require analogous TBE prevention to the healthy population of their region.

Therefore, the prevalence of protective anti-TBE IgG levels in PAD patients under IgG replacement therapy was assessed in this study in order to develop a strategy for patient care in individuals at high risk to TBE exposure.

## Material and Methods

### Study cohort

Analysis was performed in serum samples collected between 2003 and 2008 and supplemented by samples from 2014 from recently approved IgG products. One hundred ten patients with diagnosed PAD according to the ESID definitions^1^ were included after signing informed consent according to ethical approval (vote number 239/07, Ethics Committee University Medical Center Freiburg). All patients received regular intravenous or subcutaneous IgG replacement therapy without recent change of brand. Additional information about patient history (such as history of tick bite, meningitis in general, and previously performed TBE vaccination) and on B-cell phenotype according to Freiburg classification (4) and Euro-Class (5) was recorded. In patients receiving IVIG therapy blood samples obtained immediately prior to infusion provided trough level values, others were indicated as non-trough level measurements. Serum samples were analyzed both retro- and prospectively. In 8 patients, TBE titers were determined under two and in 2 patients under three different Ig preparations leading to a total of 122 cases of patient-preparation combinations. In 29 of the 122 cases, two or more consecutive measurements were performed over time to test for reproducibility.

### Laboratory assessments

Total serum IgG was measured by nephelometry using standard test kits (Dade-Behring kit, BN II nephelometer).

The SERION ELISA classic TBE Virus IgG test is a qualitative and quantitative immunoassay for the detection of human antibodies in serum, plasma, or cerebrospinal fluid directed against TBE viruses. The antibody activity is expressed in units per milliliter with a cut-off at 150 U/ml and a grey zone between 100 and 150 U/ml. The evaluation of the IgG antibody activity is referenced to the first standard serum for human IgG antibodies against TBE Virus of the Consultant Laboratory for TBE Viruses located at the Robert Koch Institute (RKI) in Berlin, Germany. Samples collected before 2013 were measured with an earlier version of the immunoassay with a different unitation. According to the manufacturer, a factor of 5.6 may be used for conversion of old values in new standard values. All results are therefore expressed in units per milliliter according to the new RKI standard. For details of the methods, we refer to the manufacturer’s information.

The recombinant ED3 immune complex ELISA was performed as described before (6). Briefly, microtiter plates were coated with rheumatoid factor IgM (10 g/ml PBS + 1 mg/ml NaN3). The plates were blocked (1 h; 10 mg/ml bovine serum albumin in PBS) and washed (Tris-Tween) and stored at −80°C before use. Human sera were diluted 1:10 in PBS + 2% BSA. In each assay, two standard positive and three negative control samples were included. To 25 μl of diluted serum, 25 μl of peroxidase-labeled ED3 antigen, diluted 1:20,000, were added. The plates were incubated at 4°C over night. After washing 50 μl TMB (tetramethylbenzidine) substrate was added to each well and the reaction was stopped after 10 min by adding 50 μl of H_2_SO_4_ stopping solution. The reaction is read at 450 nm. The cut-off of the assay is at 0.150 OD with a gray zone between 0.100 and 0.150 OD. Results are calculated by dividing the OD of the sample by 0.150 and expressed as Signal/Cut-off (S/Co).

### IgG products

Data on the origin of plasma donors were taken from the respective product information or were provided by courtesy of the companies. Epidemiological data concerning vaccination frequencies among different European populations have been reported for 2007–2009 by Kunze et al. and are listed in Table 1 (7).

**Table 1:** **Vaccination rates of different European countries 2007–2009**.

Country	Vaccination rate (%)
Austria	58 (88% incomplete)
Latvia	39
Germany	26
Estonia	20
Switzerland	17
Czech Republic	16
Sweden	13
Slovenia	13
Lithuania	10

Up to date there exists no licensed TBE vaccine in the USA^2^

Due to these aspects, plasma preparations were grouped as North American, European, or a mixture of both in this analysis (Table 2).

**Table 2:** **Origin of plasma – assignment of product codes**.

Product codes	North American	Mixture	European
IVIG	1, 2, 3, 4	5, 6, 7	8, 9
SCIG	10	11, 12	13

## Results

**Figure 1:**
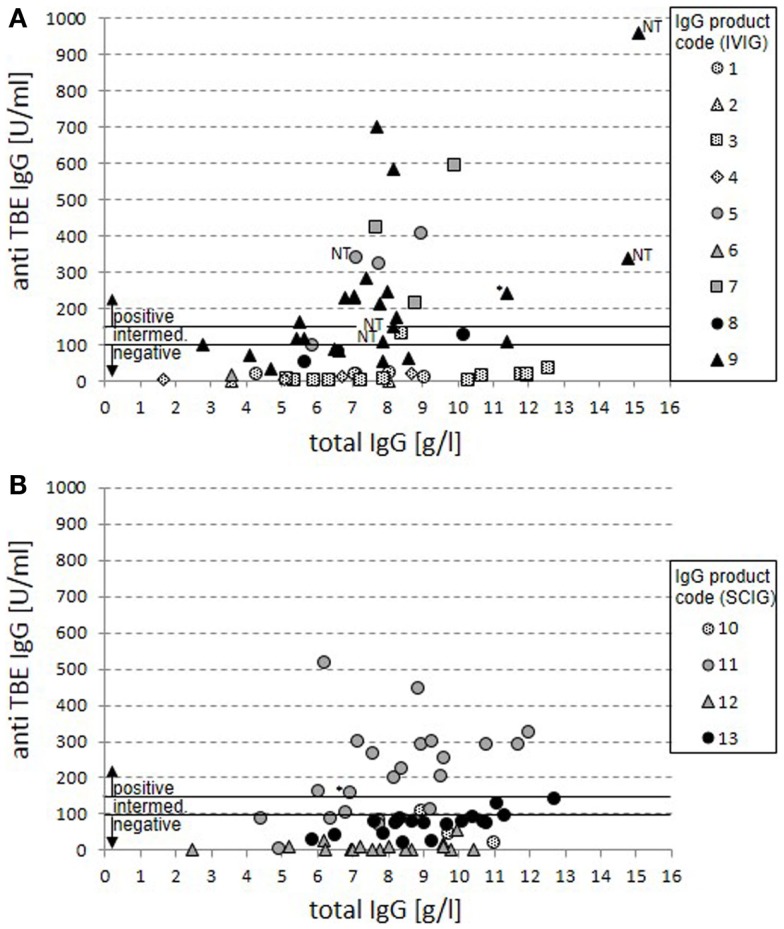
**Anti-TBE IgG vs. total IgG serum levels in patients under IgG replacement therapy**. Anti-TBE IgG titers measured by standard ELISA are shown in relation to total IgG serum levels for 60 cases from patients treated with IVIG **(A)** and 62 cases from patients treated with SCIG products **(B)**. Each case of patient-product combination is presented by a single value. Product codes are assigned to symbols with dotted filling representing North-American origin of plasma, gray filling mixed origin, and black filling European origin. The anti-TBE antibody titers for positive (>150 U/ml), intermediate (100–150 U/ml), and negative (<100 U/ml) serum titers are marked. In four cases on IVIG replacement with positive anti-TBE titers, only non-trough levels were available (NT). Positive values are also marked in two cases where a sustained active anti-TBE antibody response cannot be excluded because of normal switched memory B cells and vaccination (*).

Anti-TBE-specific IgG values were measured by a standard ELISA (Serion) in relation to total IgG levels for IVIG (Figure 1A) and for SCIG preparations (Figure 1B). Overall, 75 negative, 12 intermediate, and 35 positive results were detected with 33 negative, 7 intermediate, and 20 positive cases for IVIG preparations (Figure 1A) and 42 negative, 5 intermediate, and 15 positive cases for SCIG preparations (Figure 1B).

Products with North-American origin never achieve anti-TBE-specific IgG levels above 150 U/ml in patients (25 negative and 2 intermediate cases). For both mixed and European products in 35 out of 95 cases, positive anti-TBE titers can be detected (compared to 50 negative and 10 intermediate results). There were clear differences between single products in this group revealing the highest titers of anti-TBE antibodies in the IVIG products 5, 7, and 9 and the SCIG product 11, while there were no truly positive serum titers achieved in patients on products 8, 12, and 13. Product 6 was applied only in one case with insufficient IgG serum trough levels at the point of measurement and can therefore not be judged.

Based on our previous data (4, 8), in two cases with positive anti-TBE titers, a sustained active anti-TBE vaccination response could not be excluded because of the combination of normal switched memory B-cell counts and a history of vaccination against TBE virus.

**Figure 2:**
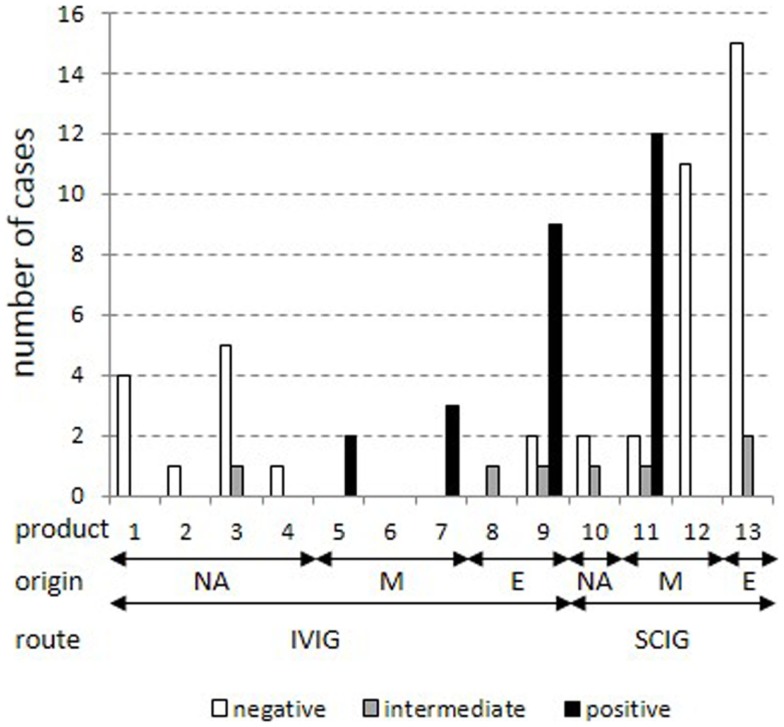
**Stratification of cases for each product in patients with IgG trough levels above 7 g/l**. All cases with total IgG trough levels above 7 g/l were stratified into positive, intermediate, and negative results in the standard anti-TBE ELISA according to IgG products as coded in Table 2. Plasma origin is indicated as North America (“NA”), mixture (“M”), and Europe (“E”).

The distribution of cases was analyzed according to the anti-TBE response for each of the 13 products (Figure 2). Cases were restricted to cases with IgG trough levels above 7 g/l. This confirmed the previously identified four products with mostly positive anti-TBE serum levels (5, 7, 9, and 11) and eight products with mostly negative/intermediate anti-TBE serum levels (1, 2, 3, 4, 8, 10, 12, and 13). Again, all North-American products, but also some purely European products (8 and 13) were negative, reflecting the regional heterogeneity of the vaccination status against TBE in Europe. Unfortunately, more detailed information on the composition of mixed and European products regarding the specific country of origin and the particular mixture of single plasma batches is not routinely available through the producing companies.

**Figure 3:**
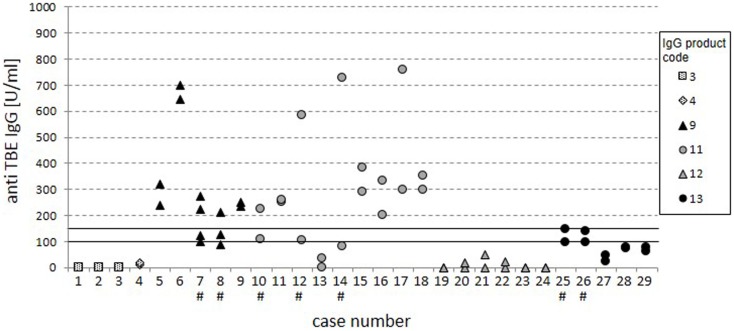
**Cases with consecutive measurements**. The dot plot displays anti-TBE IgG titers from the standard ELISA for 29 separate cases with consecutive measurements. Cases with inconsistent ratings are highlighted by “#.” The variation of specific anti-TBE IgG titers was not due to variation in total IgG serum levels.

To assess the reproducibility of the results, consecutive trough serum samples within 29 cases were analyzed for global anti-TBE titers (Figure 3). Twenty-two cases showed stable measurements at different time points with consistently negative or positive results. At overall grading in negative, intermediate, and positive results, five cases (7, 8, 10, 25, and 26) had values in the intermediate region and in two cases (12 and 14) the titers clearly varied (indicated by #), which is not explained by parallel variations in total IgG serum levels (data not shown).

**Figure 4:**
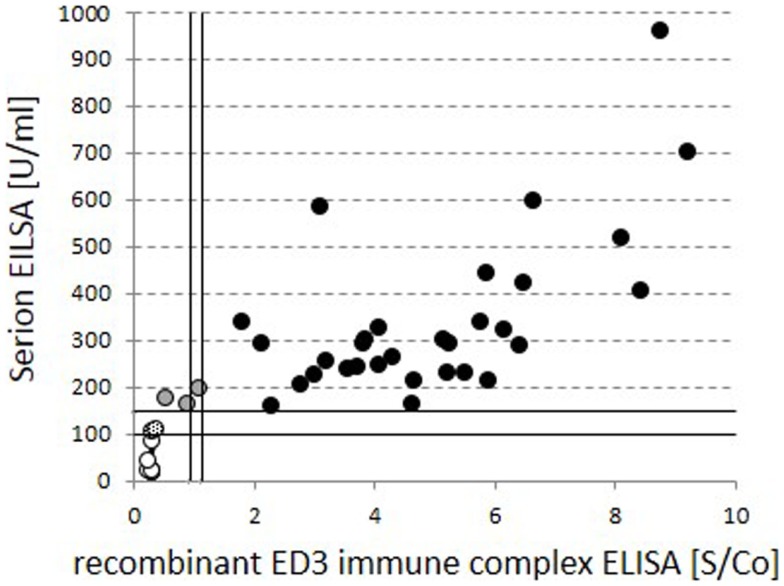
**Comparison of global vs. neutralizing anti-TBE IgG titers**. Quantitative test results for global standard anti-TBE IgG titers from the standard ELISA are compared to neutralizing, anti-recombinant ED3 immune complex ELISA values within identical serum samples. Circles filled in black indicate positive and open circles indicate negative results in both tests. Gray and dotted fillings reflect deviant grading within the two assays.

Standard anti-TBE IgG ELISAs are known to have limited informative value in regard to actual protection. More informative assays detecting neutralizing antibodies on the other hand are currently rarely performed and more elaborate. We confirmed the findings of the standard ELISA in a selection of 46 cases in a test for neutralizing antibodies with an ELISA for anti-ED3 of TBE antibodies (Figure 4; Table 3). Our data show that 31 out of the 35 positive results from the standard ELISA could be confirmed by the detection of neutralizing antibodies. Standard ELISA results in the lower positive range (150–200 U/ml) changed to intermediate or even negative in three cases or were confirmed as positive in another three cases. Standard ELISA results above 200 U/ml were confirmed positive for neutralizing antibodies in all cases. Single random samples from intermediate or negative results in the standard ELISA always turned out to be negative for neutralizing antibodies as expected (Table 3).

**Table 3:** **Comparison of standard ELISA for anti-TBE IgG vs. neutralizing antibodies**.

Serion ELISA result	Neutr. Ab neg	Neutr. AB intermed.	Neutr. AB positive	Neutr. AB not tested
75 negative	10	0	0	65
12 intermediate	2	0	0	10
35 positive	1	2	31	1

## Discussion

Due to disease-associated impairment of vaccination responses, patients with primary antibody deficiencies depend on the quality and quantity of passive protection via the application of polyvalent IgG preparations. For most diseases including TBE, which can be prevented by vaccination, the induced protection is provided via antibodies. The impact of geographic plasma origin on the presence of distinct specific IgG titers within polyvalent IgG preparations has been described before. Rabel et al. had already reported geographic differences for TBE within IVIG products (2). In parallel, Seidel et al. found divergent levels of protective antibodies in a small number of patients with IgG replacement therapy but did not correlate their findings to the geographic plasma origin in detail (3). Our data complement the previous results by analyzing TBE-specific serum titers in patients in relation to the geographic origin of the received IgG preparation.

An epidemiological proof of protection against TBE infection is impossible given the small number of CVID patients under substitution, but we could show a good correlation of high anti-TBE IgG titers in the global ELISA (>200 U/ml) and the presence of anti-ED3 neutralizing antibodies. Antibodies directed to the ED3 protein of TBE virus are highly specific for TBE virus without cross reactivity to other flaviviruses and have shown strong neutralizing activity and are thought to be protective against TBE (6, 9–11). In contrast, reactivity in global anti-TBE ELISAs using lysate of cell cultured virus, may not always be due to neutralizing antibodies and cross reactivity with other flaviviruses, e.g., yellow fever virus or dengue virus cannot be excluded (12). Our data demonstrate strong differences in TBE-specific IgG titers among patients under IgG substitution depending on the origin of plasma. In 35 patients, anti-TBE titers were positive with positive evidence of protective neutralizing antibodies in most cases.

All purely North-American products were consistently negative being in line with the previously published findings from measurements within IVIG products (2). The results for mixed and European products were variable with 26 positive, 5 intermediate, and 30 negative results looking at cases with total IgG trough levels above 7 g/l. This variability among IgG preparations with European plasma contribution was highly dependent on the specific product. Most patients under replacement with products 5, 7, 9, and 11 had positive results, while serum samples from patients with products 8, 12, and 13 were negative or intermediate. It is tempting to speculate that the content of North-American plasma is of major influence in the mixed group. However, given the variability within purely European-derived plasma products, it becomes clear that the country of origin within Europe has a similarly strong influence as disclosed already by the different vaccination policies in Table 1. Even within a given product variable, anti-TBE serum titers were found as exemplified by product 9 suggesting that over time the composition of different batches varied in regard to origin, mixture, and obviously specific donors within the countries. Due to lack of detailed information on the relative composition of the batches applied currently treating physicians have no chance to predict the presence of protective levels for specific products. This would not only require the disclosure of the composition of the specific batches by the plasma-producing companies but also a better estimate of an average value of national anti-TBE titers in European countries, which may vary according to changing policy over time and therefore is not feasible.

In daily practice, we suggest physicians to consider the patient’s personal risk for specific infection, in this case TBE, as one aspect when selecting an appropriate IgG preparation at the initiation of IgG replacement therapy.

For patients already on IgG replacement therapy, we recommend the following approach for patients at high risk for TBE exposure:

A) Patients receiving IgG products with definitive exclusive plasma origin from North America: no anti-TBE IgG testing is necessary. The patient needs to be informed, that no passive protection can be expected. Other types of prophylaxis need to be emphasized. Whether a change of product with the option of a passive protection is justified has to be made by individual decision.
B) All others patients: standard anti-TBE ELISA testing at the time of trough levels is suitable. In serum samples with anti-TBE titers above 200 U/ml, the presence of protective neutralizing antibodies can be assumed. However, patients need to be informed about possible variations due to changes in batch composition. In case of negative or intermediate result in high-risk patients, such as lumberjacks in endemic areas, it might be helpful to contact the producing company for disclosure of the definite composition of the relevant batch. This should be added by the information whether in this individual case the patient could be provided with selective batches containing higher amounts of plasma from distinct European countries with better TBE vaccination rates.

However, this procedure still bears uncertainties due to variation between single batches of the same product. Therefore, we recommend plasma-producing companies within the European market to add measurements of anti-TBE IgG within each charge and to provide physicians treating PAD patients with these data. Alternatively, the display of a defined code within the batch numbers for the countries of plasma origin might enable treating physicians to choose products based on the national vaccination policies described above.

These data emphasize the importance of regional plasma donation to address the diverse local infection profiles in a better way. Patients with PAD lacking specific antibody responses depend on the broad passive protection of the IgG preparations provided by plasma-producing manufacturers. Optimal vaccination status of the plasma donors according to official regional recommendations can greatly contribute to the quality of the product.

## Conflict of Interest Statement

Sigune Goldacker: Honoraria from Baxter, research grant from Octapharma. Torsten Witte: Honoraria from CSL Behring and Octapharma. Hans-Hartmut Peter: Member of the Pfizer scientific advisory board of the pneumococcal conjugate vaccine PCV13 program in Germany and honoraria from CSL Behring and Novartis. Klaus Warnatz: Honoraria from Baxter, CSL Behring, Octapharma, Biotest, research grant from Baxter. Daniela Huzly and Michael Schlesier have no conflicts of interest to declare.

## Footnotes

^1^http://www.esid.org

^2^http://wwwnc.cdc.gov/travel/
